# Evaluation of Antioxidant Systems and Ascorbate-Glutathione Cycle in Feijoa Edible Flowers at Different Flowering Stages

**DOI:** 10.3390/foods9010095

**Published:** 2020-01-16

**Authors:** Anna Magri, Giuseppina Adiletta, Milena Petriccione

**Affiliations:** 1 Consiglio per la Ricerca in Agricoltura e l’Analisi dell’Economia agraria (CREA), Centro di ricerca Olivicoltura, Frutticoltura e Agrumicoltura, Via Torrino 3, I-81100 Caserta, Italy; annamagri93@gmail.com; 2 Department of Industrial Engineering, University of Salerno, Via Giovanni Paolo II, 84084 Fisciano, Italy; gadiletta@unisa.it

**Keywords:** edible flowers, feijoa flowers, antioxidant compounds, ascorbate-glutathione cycle

## Abstract

Background: Feijoa (*Acca sellowiana* (O. Berg)) was initially introduced as an ornamental plant, but nowadays, it is widely cultivated for the numerous beneficial properties of its edible fruits. Feijoa flowers have been included in the list of edible flowers, but despite this, they are still considered niche products due to consumer skepticism and lack of publicity. Methods: This study evaluated the physicochemical traits, antioxidant system, and ascorbate-glutathione cycle in feijoa flowers at five flowering stages based on the *Biologische Bundesanstalt, Bundessortenamt und CHemische Industrie* (BBCH) scale. Results: The results showed that the optimal stage to harvest feijoa flower is the F2 stage characterized by high bioactive compounds content. Furthermore, the enzymes involved in oxidative stress and the ascorbate-glutathione cycle showed different trends during the flowering stages. Conclusions: This study provides new evidence to understand the possible role of bioactive compounds and ascorbate-glutathione cycle in the regulation of flower development, defining the optimal stage to harvest flowers.

## 1. Introduction

Feijoa (*Acca sellowiana* (O. Berg)) belongs to the *Myrtaceae* family. The feijoa trees have Columbian origin, but they are well-adapted to other regions, such as New Zealand, Italy, France, Turkey, and Iran [1]. This species was introduced as an ornamental plant, but nowadays, is widely cultivated for its edible fruits with numerous beneficial properties [2]. However, fruits are not the only edible part; indeed, the petals of hermaphrodite showy flowers are too. Flowers bear a single carpel and numerous stamens (60–90), which are bright crimson red with white-pink fleshy petals. The petals show a form from suborbicular to elliptic and their numbers range from four to six with a fleshy texture and sweet taste. Indeed, they can be used in salads, infusions, or as food decorations [3,4]. Edible flowers have been used in various ancient cultures such as Greece, Rome, India, and China for their beneficial, nutritional, and medicinal properties [5,6]. An official list of edible flowers has been emitted by the Food and Agriculture Organization of the United Nations (FAO) and the World Health Organization (WHO). Nowadays, edible flowers are also considered niche products, although several studies have been reported their health benefits [7]. To promote their consumption, as a common food, it is necessary to develop nutritional education [8]. The number of edible flowers varies in different countries and they are obtained from 97 families, 100 genera, and 180 species worldwide [9]. Depending on the species, it is possible to eat whole flowers, flower buds, or some parts such as petals and pistils. The inflorescences of artichoke, broccoli, and cauliflower are daily consumed as vegetables in the human diet, while the flowers of fruit trees, such as elderberry or citrus, are used to prepare syrups [5]. Nowadays, consumers are searching constantly for new sources of antioxidants that are beneficial against premature aging, metabolic, chronic, cardiovascular, Alzheimer diseases, and cancer [10]. Several studies have revealed that antioxidant activity in edible flowers is due to phenolic compounds (including phenolic acids, flavonoids, anthocyanins, tannins), carotenoids, and vitamins such as tocopherols and ascorbic acid [11]. These secondary metabolites determine a wide variability of colors in edible flowers that, combined with volatile compounds, contribute to increasing consumer acceptability [12]. Different phenolic compounds have been identified in several botanical parts of feijoa such as flower buds, fruits, and leaves [13,14,15]. Pedunculagin, gallic acid, hyperoside, gossypetin arabinofuranoside, ellagitannin, ellagic acid, and several anthocyanins have been identified in flower buds [13]. Among all identified compounds, ellagic acid is the most abundant phenolic compound in flower buds with a concentration of 76 µg/10 mg of extract [13]. These bioactive compounds change during flower development and senescence, and several biochemical and molecular pathways are activated to counteract the increase of reactive oxygen species (ROS) and free radicals. Only limited literature data are available on the antioxidant compounds changes and their biological properties in ornamental flowers during the flower development process until the senescence, but data on the enzymatic antioxidant system is unavailable in edible flowers [16]. The aim of this study was to evaluate the physicochemical traits and bioactive compounds content in feijoa flowers at five developmental stages. Furthermore, antioxidant enzymes involved in the regulation of the ascorbate-glutathione cycle to maintain redox status during flower opening have been evaluated. 

## 2. Materials and Methods 

### 2.1. Flower Samples and Chemicals

**Figure 1:**
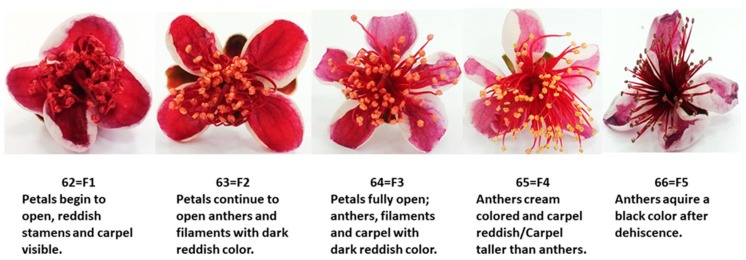
Different flowering stages of feijoa flowers. Petals begin to open: Reddish stamens and carpel visible—F1; petals continue to open: Anthers and filaments with dark reddish color—F2; petals fully open: Anthers, filaments, and carpel with dark reddish color—F3; anthers cream-colored and carpel reddish/Carpel taller than anthers—F4; anthers acquire a black color after dehiscence—F5.

Feijoa flowers (cv. *Mammouth*) were randomly harvested at five flowering stages from different trees grown in the experimental orchard, located in Caserta (Caserta, Southern Italy) and owned by the CREA-OFA. Based on the BBCH scale [17], the stages corresponding to 62 (petals begin to open; reddish stamens and carpel visible—F1), 63 (petals continue to open; anthers and filaments with dark reddish color—F2), 64 (petals fully open; anthers, filaments and carpel with dark reddish color—F3), 65 (anthers cream-colored and carpel reddish/Carpel taller than anthers—F4), and 66 (anthers acquire a black color after dehiscence—F5) values were chosen for this study (Figure 1). Flowers were transported to the laboratory and petals were manually separated from flowers and stored at −20 °C until analysis. Three biological replicates were collected for each flowering stage and each replicate contained about 150 petals from 30 flowers. Experimental analyses were carried out from three technical replicates for each biological replicate.

All chemicals used were of analytical grade and were purchased from Sigma-Aldrich (Milano, Italy).

### 2.2. Physicochemical Properties

The color of the inner petal side was measured using a Minolta colorimeter (Minolta C2500. Konica Minolta, Ramsey, NY) to define chromaticity values L* (Lightness), a* (green to red), and b* (blue to yellow). Chroma (CHR) and hue angle (HUE) were calculated from a* and b* values. 

Titratable acidity (TA), expressed as g of citric acid equivalent per 100 g of fresh weight (g citric acid 100 g^−1^ of FW), was determined by acid-base titration using a digital pH meter (Model 2001, Crison, Barcelona, Spain). The pH value of each flowering stage was estimated on flower juice using the same digital pH meter at 20 °C [18]. 

Reducing sugars were determined by Fehling assay, according to previously established methodologies [19]. The results were expressed in g of reducing sugars in 100 g of fresh weight (FW). 

### 2.3. Bioactive Compounds and Antioxidant Activity

The extract to determine total phenolic and flavonoid content was obtained after homogenizing petals (2.5 g) in a water-alcohol solution (methanol/water 80:20 *v/v*). The homogenize was centrifuged at 14,000× *g* for 5′ at 4 °C and filtered through Whatman No. 41 paper. The supernatant was used for the analysis. The total phenols (POL) and the total flavonoids (FLAV) contents were determined by the Folin–Ciocalteu reagent and aluminum chloride colorimetric method reported by Goffi et al. [20]. POL and FLAV were expressed as milligrams of gallic acid equivalents (GAE) 100 g^−1^ of FW and milligrams of catechin equivalent (CE) 100 g^−1^ of fresh weight (FW), respectively. 

Free radical scavenging activity was evaluated from homogenized petals (5 g) in a water-alcohol solution (methanol/water 80:20 *v/v*). The homogenized was centrifuged at 10,000× *g* for 10′ at 15 °C. The hydroalcoholic extract was used to evaluate antioxidant activity. The free radical scavenging activity was measured by 1,1-diphenyl-2-picryl-hydrazil (DPPH) according to Adiletta et al. [21], with some modifications. The assay reaction contained 50 µL of extract and 6.25 × 10^−5^ M solution of DPPH dissolved in methanol (1.450 mL), and the decrease of absorbance was evaluated at 515 nm after 10 min of incubation. The antioxidant activity was expressed as µmol Trolox equivalent (TE) g^−1^ of FW. 

The ascorbic acid content from feijoa petals was measured following the method of Petriccione et al. [22], with some modifications. Petals (2.5 g) were blended using 10 mL of 16% (*v/v*) metaphosphoric acid solution containing 0.18% (*w/v*) disodium ethylene diamine tetraacetic acid. The mixture was centrifuged at 11,000× *g* for 10 min at 4 °C. Assay reaction contained 400 μL of extract, diluted Folin’s reagent (5:1, *v/v*), and 0.3% metaphosphoric acid (*v/v*). The absorbance of the extract was registered at 760 nm with a UV-VIS Spectrophotometer (Model V-630, Jasco, Tokyo, Japan). The ascorbic acid content was expressed as mg ascorbic acid (AA) 100 g^−1^ of FW. 

The total monomeric anthocyanins were estimated spectrophotometrically at 510 and 700 nm and expressed as mg of cyanidin-3-glucoside equivalent (CGE) 100 g^−1^ of FW. 

### 2.4. Antioxidant Enzymes

Total soluble proteins were extracted by homogenizing frozen petals powder (1:3 *w/v*) in buffer containing 0.1 M of potassium phosphate (pH 7), 1 mM of sodium-ethylenediaminetetraacetic acid (EDTA pH 7), 6.25 mM of polyethylene glycol (PEG), 5% (*w/v*) polyvinylpolypyrrolidone (PVPP), and 5 mM of ascorbic acid (only for APX enzyme extraction). The mixture was centrifuged at 14,000× *g* for 20 min at 4 °C. The supernatant was used to measure the total soluble protein content by the Bradford assay [23], catalase (CAT), superoxide dismutase (SOD), glutathione reductase (GR), dehydroascorbate reductase (DHAR), monodehydroascorbate reductase (MDHAR), and ascorbate peroxidase (APX) activities.

#### 2.4.1. Catalase and Superoxide Dismutase Activity 

Catalase (EC 1.11.1.6) activity was evaluated by the method of Goffi et al. [24], with minor modifications. Reaction mixture included 100 mM of potassium phosphate buffer (pH 7), 20 mM of H_2_O_2_, and 20 µL of crude enzyme extract. The reaction was monitored with a decrease in absorbance at 240 nm. CAT activity was expressed in µmol of H_2_O_2_ g^−1^ of FW. 

Superoxide dismutase (EC 1.15.1.1) activity was evaluated according to Petriccione et al. [25], with minor modifications. The reaction mixture included 50 mM of potassium phosphate buffer pH 7.8, 0.1 mM of sodium EDTA, 13 mM of methionine, 75 µM of nitro blue tetrazolium chloride (NBT), 0.5 µM of riboflavin, and 50 µL of crude enzyme extract. The reaction was produced by adding riboflavin. The absorbance was measured after 10 min of incubation at room temperature under continuous light at 560 nm. One SOD unit was expressed as the amount of enzyme that inhibits the rate of NBT reduction by 50% below the assay conditions. SOD activity was expressed as U g^−1^ of FW. 

#### 2.4.2. Ascorbic Acid Metabolism

Ascorbate peroxidase (EC 1.11.1.11) activity was measured by the method of Pasquariello et al. [26], with some modifications. Assay reaction included 100 mM of potassium phosphate buffer (pH 7), 0.33 mM of ascorbic acid, 0.35 mM of H_2_O_2_, 0.66 mM of sodium EDTA (pH 7), and 50 µL of crude enzyme extract. The oxidation of ascorbic acid was read at 290 nm and APX activity was expressed as µmol of ascorbate g^−1^ of FW.

Monodehydroascorbate reductase (EC 1.6.5.4) activity was assayed spectrophotometrically by the method of Arrigoni et al. [27], with minor modifications. The assay was performed with a reaction mixture containing 50 mM of Tris(hydroxymethyl)aminomethane hydrochloride with a pH of 7.6 (Tris-HCl), 2.5 mM of sodium ascorbate, 0.1 mM of reduced disodium salt hydrate (NADH), 0.25 U of ascorbate oxidase, and crude enzyme extract. The absorbance was measured at 340 nm by monitoring NADH oxidation and was expressed in µmol s^−1^ kg^−1^ of FW. One unit is the amount of enzyme that oxidizes 1 nmol of NADH per min. 

Dehydroascorbate reductase (EC 1.8.5.1) activity was evaluated according to Asada (1981) [28], with slight modification. The reaction mixture contained 50 mM of potassium phosphate with a pH of 7, 0.1 mM of sodium EDTA with a pH 8, 2.5 mM of reduced glutathione, 0.2 mM of dehydroascorbate, and crude enzyme extract. The absorbance was measured at 265 nm by monitoring the increase of ascorbate and was expressed in µmol s^−1^ kg^−1^ of FW. 

Glutathione oxidoreductase (EC 1.6.4.2) activity was measured following the method of Garcia-Limones et al. [29], with some modifications. The reaction mixture contained 50 mM of potassium phosphate with a pH of 7, 0.5 mM of glutathione disulphide (GSSG), crude enzyme extract, and 0.2 mM of dihydronicotinamide-adenine dinucleotide phosphate (NADPH). The absorbance was recorded at 340 nm for 300 s and was expressed in µmol s^−1^ kg^−1^ of FW.

#### 2.4.3. Polyphenoloxidase Activity 

Polyphenoloxidase activity (EC.1.10.3.1; PPO) was measured with the method described by Modesti et al. [30]. Crude enzyme extract was derived by homogenizing petals (1:3 *w/v*) in sodium phosphate buffer (100 mM, pH 6.4) and 0.05 g of PVPP. PPO activity was evaluated at 398 nm using 500 mM of catechol dissolved in 100 mM of sodium phosphate buffer with a pH 6.4 and 100 µL of crude enzyme extract. PPO activity was expressed as µmol g^−1^ FW.

### 2.5. Statistical Analysis 

All data were expressed as the mean ± standard deviation. Statistical significance in feijoa flowers harvested at five flowering stages was analyzed by analysis of variance (ANOVA) and Duncan’s Multiple Range test at a 5% level to compare the differences between means. Principal components analysis (PCA) was applied to obtain new components that account for most of the variation in the original data with a reduction in dimensionality, evaluating the physicochemical, qualitative, and enzymatic traits during flowering stages. Correlation coefficients between different traits were evaluated by Pearson analysis. Statistical analysis was carried out using the SPSS software package, Version 20.0 (SPSS Inc., Chicago, IL, USA).

## 3. Results and Discussion 

### 3.1. Physicochemical Traits

The physicochemical traits are important indicators that reflect the palatability and quality of flowers. Reducing sugars (RS) content displayed an increasing trend due to high metabolic activity during flower opening, while titratable acidity (TA) decreased. At the F5 flowering stage, feijoa petals showed a very low acidity (0.04 citric acid g/100 g of FW), which correlated to a subneutral pH (6.48) and a high reducing sugars content (4.84%). Our results were confirmed by Dettori and Di Gaetano [31] who demonstrated that feijoa petals have a high sugar content in different developmental stages because they did not produce nectar. Wide variability has been found in acidity and sugar content in several edible flowers such as *Bauhinia variegata* [32], *Allium schoenoprasum*, *Rauvolfia micrantha*, and *Yucca filifera* [5,33]. Color is an important qualitative trait and it changes during flower development, as reported by Schmitzer et al. [34]. Edible flowers are perishable and petal color is an important parameter susceptible to tissue browning due to loss of cell compartmentalization and enzymatic reactions that involve several bioactive compounds. During flower development, the inner color of petals changed as follows: F1, Persian Plum; F2, Antique Ruby; F3, Claret; F4, Quinacridone Magenta; and F5, Twilight Lavender (Figure 1), with chroma and hue angle values ranging from 50.24 (F1) to 24.77 (F5) and from 23.54 (F1) to 355.13 (F5), respectively (Table 1). The visual observation of petal color (Figure 1) was in good agreement with the color obtained from the calculated L*, a*, and b* coordinates (Table 1). Color changes are probably due to the specific role of photosynthetically active chloroplasts in flower development in anthocyanin-rich reddish petals as suggested by Katz and Weiss [35].

### 3.2. Bioactive Compounds and Antioxidant Activity

**Figure 2:**
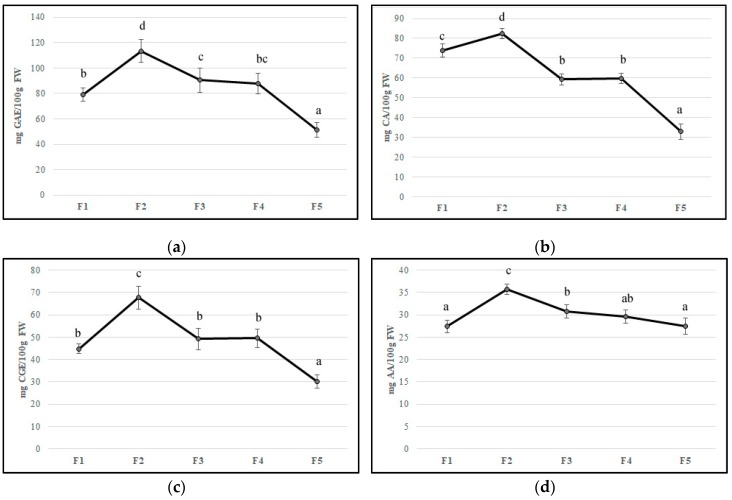

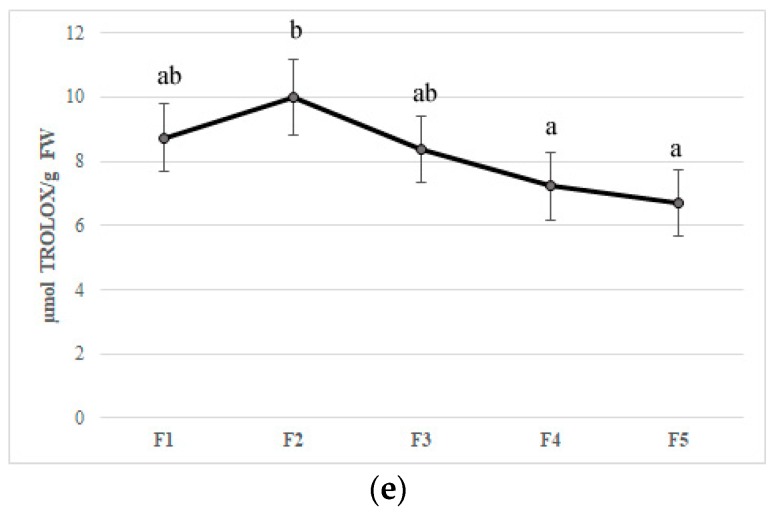
Bioactive compounds content (**a**) polyphenols, (**b**) flavonoids, (**c**) anthocyanins, (**d**) ascorbic acid, and (**e**) antioxidant activity in five different flowering stage of feijoa (F1, F2, F3, F4, F5). Means followed by the same letter do not differ significantly at *P* = 0.05 (Duncan Test).

Flower development is accompanied by a substantial change in concentrations of bioactive compounds and petal color [34]. In the edible component of flowers can be found molecules with beneficial health properties such as polyphenols, anthocyanins, ascorbic acid, and flavonoids that can be found in edible flowers. Their important roles in counteracting oxidative stress involved in chronic disease development have been reported [36,37,38]. In our study, the F2 stage showed the highest values of all bioactive compounds, probably due to stress caused by flower development. The total phenolic content decreased during feijoa flower development with values from 79.28 mg GAE/100 g of FW (F1) to 51.42 mg GAE/100 g of FW (F5), with the highest values at F2 (113.4 mg GAE/100 g of FW) (Figure 2A). Our results are in agreement with Stefaniak and Grzeszczuk [39], who demonstrated that POL content changed in different species of edible flowers with values ranging from 2.06 to 4.33 mg GAE/g of FW. Anthocyanins and flavonoids are responsible for the red-violet color of flowers acting as insect and animal attractants [40]. The characteristic structure of flavonoids allows the scavenging of free radicals and quenching of oxygen singlet, inhibition of lipoxygenase that can cause lipid oxidation [37]. The number of anthocyanins and flavonoids decreased during floral stages with a peak in F2, as found for TP content (Figure 2B,C). Anthocyanins values agree with Benvenuti et al. [38], who analyzed several red and pink edible flowers. Then, a decline in the concentration of phenolic compounds at the end of flower development made the flowers more vulnerable to oxidative stress. The concentration of total anthocyanins in petals that declined during flower development is also reported in *Rosa hybrida* ‘Happiness’ and ‘Pink Pink Coronet’ [41], *Petunia hybrida* Hort. [42], and *Hydrangea macrophylla* Thunb. [43]. Several studies have demonstrated that the decrease of anthocyanin content might be due to different factors such as active degradation of this pigment, dilution of pigments consequently to petal expansion, and cessation of petal expansion at the final stages of development [42,44]. As suggested by Schmitzer et al. [39], the process of senescence is linked to the anthocyanin concentration in rose flowers. Noda and co-workers [45] suggested that a sequential biosynthesis of flavanols and anthocyanins occurs in *Eustoma grandiflorum* during floral development, while Dong et al. [46] demonstrated a decline in phenylalanine ammonia-lyase (PAL), chalcone isomerase (CHI), and dihydroflavonol 4-reductase (DFR) gene transcription in apple flowers. The trend of anthocyanins content in feijoa flower development could be linked to a color that changes from red to pink-violet during senescence [41]. Ascorbic acid content reached the highest value in F2 (35.76 mg per 100 g of FW); afterward, it decreased up to 27.5 mg per 100 g of FW (F5) (Figure 2D). Our results are in agreement with Cavaiuolo et al. [47], who indicated an increase in the ascorbic acid content during early senescence, followed by a decrease in advanced senescence. The antioxidant activity, measured by DPPH activity and expressed in Trolox equivalent, was correlated with bioactive compound trends. A peak was observed in F2 petals with a subsequent decrease up to F5 (Figure 2E). Different studies reported that bioactivity of edible flowers is highly correlated to bioactive compounds especially to phenolic compounds composition [48,49,50,51]. Significant differences in both phenolic compounds and antioxidant activity between edible flowers have been found [49]. Chen et al. [50] demonstrated that *Rosa rugosa* Thunb. (purple) and *Rosa rugosa* Thunb. (pink) displayed the highest DPPH (612.79 and 544.75 μmol Trolox/g DW), FRAP (273.10 and 301.14 μmol Trolox/g DW), and TEAC (1013.71 and 937.19 μmol Trolox/g DW) values among thirty tested flowers. Furthermore, gamma and electron-beam irradiation used as a postharvest treatment in edible flowers improved the phenolic content and antioxidant activity [48]. 

### 3.3. Antioxidant Enzymes

**Figure 3:**
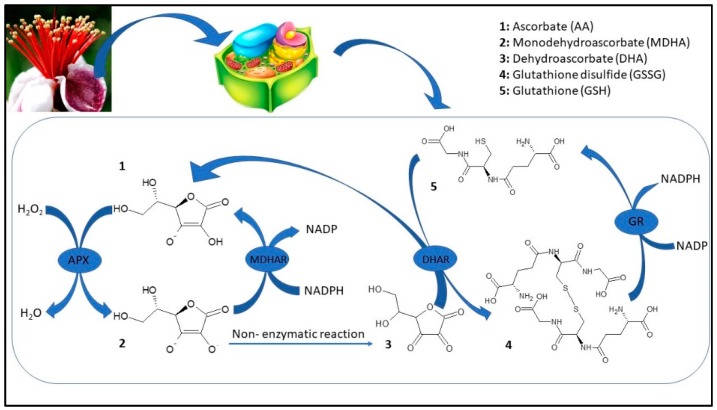
Ascorbic acid-glutathione cycle (APX: Ascorbate peroxidase; MDHAR: Monodehydroascorbate reductase; DHAR: Dehydroascorbate reductase; GR: Glutathione reductase).

**Figure 4:**
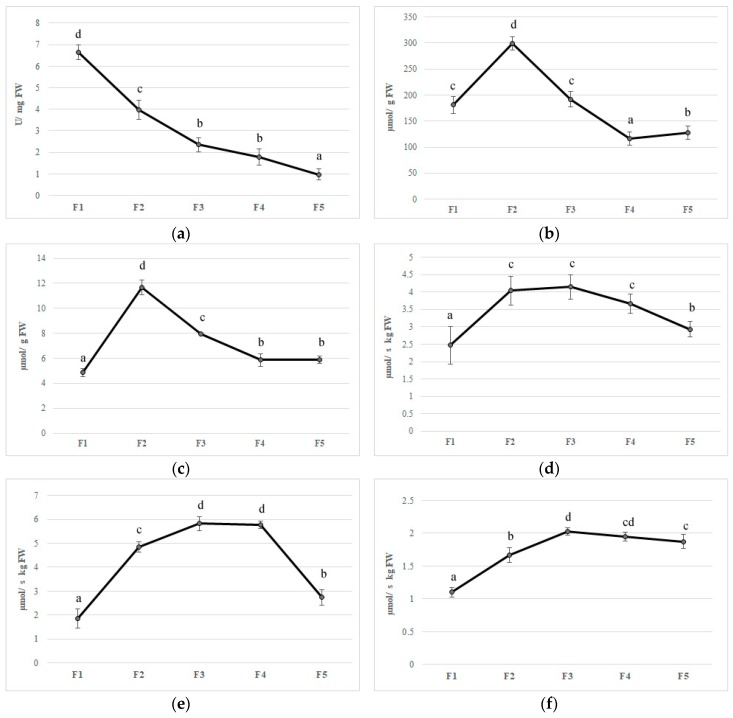

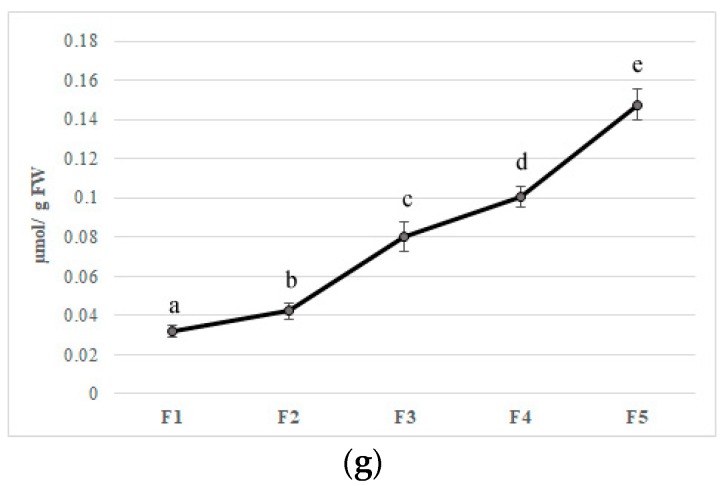
Antioxidant enzymes activities (**a**) superoxide dismutase, (**b**) catalase, (**c**) ascorbate peroxidase, (**d**) monodehydroascorbate reductase, (**e**) dehydroascorbate reductase, (**f**) glutathione reductase, and (**g**) polyphenoloxidase in five different flowering stage of feijoa (F1, F2, F3, F4, F5). Means followed by the same letter do not differ significantly at *P* = 0.05 (Duncan Test).

Feijoa flower opening has a short lifespan, of about eight days, and it is a scalar process where not all flowers open at the same time [4]. The reactive oxygen species (ROS) are generally toxic for plants and they can cause damage to different biomolecules [52]. Plants have developed an efficient defense system such as different antioxidant enzymes to contrast this oxidative stress [52]. The first antioxidant enzyme that is involved in ROS detoxification is SOD, which catalyzes the dismutation of O_2_ to H_2_O_2_. Furthermore, CAT converts H_2_O_2_ to oxygen and water; at the same time, APX, using ascorbate as an electron donor, reduces H_2_O_2_ to water. Although ROS have been considered as toxic molecules, they have an important role as signaling molecules involved in flower opening [53]. In the first stage (F1), superoxide radicals are generated by a high SOD activity; afterward, the activity of this enzyme decreased during the other flower development stages (Figure 4A). CAT and APX activity increased to their maximum on F2 with values 299.64 and 11.65 µmol per g of FW, respectively, whereupon these enzymes decreased until a plateau was reached between F4 and F5 (Figure 4B,C). In plants, acid ascorbic content is maintained stable thanks to the acid ascorbic-glutathione cycle (Figure 3) [54]. 

In the first step of this pathway, APX reduces H_2_O_2_ to water with the generation of monodehydroascorbate (MDHA) using AA as an electron donor. MDA is both disproportionated to AA and dehydroascorbate (DHA) by non-enzymatic reaction and reduces to AsA by NAD(P)H-dependent monodehydroascorbate reductase (MDHAR). DHA reductase (DHAR), using glutathione (GSH), reduces DHA and thereby regenerates AA. The oxidized glutathione (GSSG) is then regenerated by glutathione reductase (GR), utilizing reducing equivalents from NAD(P)H. Thus, APX, in combination with the effective AA–GSH cycle, functions to prevent the accumulation of toxic levels of H_2_O_2_ [54]. MDHAR and DHAR showed a similar trend during flower development, increased faster, and kept high levels until F3 and F4 in MDHAR and DHAR, respectively (Figure 4D,E). GR increased up to F3 and subsequently maintained a constant level with an average activity of 1.90 µmol per·s^−1^ kg of FW (Figure 4). PPO is involved in the browning reactions of flowers during their development [21]. PPO activity showed an increase when all stages reached a value at F5 of 0.15 µmol/g of FW, and a negative correlation has been found with polyphenol content (r^2^ = −0.693 *p* ≤ 0.01) (Figure 4G). Synergistic activity of enzymes involved in acid ascorbic-glutathione cycle allows dissipating the excess of reducing power produced during flowering and to counteract oxidative stress due to unbalance among ROS production and scavenging [54]. Furthermore, a decrease in bioactive compounds such as phenols and anthocyanins at the final stages of flower development may limit the antioxidant defense system making the flower more susceptible to oxidative stress [55]. 

Furthermore, a decrease in bioactive compounds such as phenols and anthocyanins at the final stages of flower development induced oxidative stress that may be considered a key effector of redox signaling during flower development [55].

### 3.4. Evaluation of Different Flowering Stages by PCA

**Figure 5:**
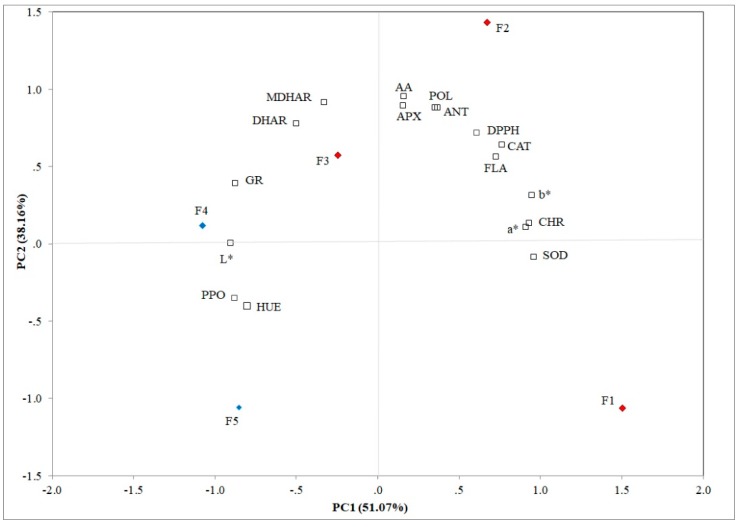
Principal component analysis of the physicochemical, bioactive, and enzymatic traits in in five different flowering stage of feijoa (F1, F2, F3, F4, F5) (POL: Total polyphenol content; AA: Ascorbic acid content; FLA: Flavonoids; ANT: Anthocyanins; DPPH: Antioxidant activity SOD: Superoxide dismutase, CAT: Catalase, PPO: Polyphenoloxidase, APX: Ascorbate peroxidase, MDHAR: Monodehydroascorbate reductase, DHAR: Dehydroascorbate reductase; GR: Glutathione reductase; L*: Lightness; CHR: Chroma; HUE: Hue angle).

PCA allows establishing the principal components (PCs) that clarified the overall variance in all analyzed traits. The first two principal components explained 89.23% of the variation (51.07% and 38.16% for PC1 and PC2, respectively) in feijoa flowers and these components showed eigenvalues ≥ 0.80 (Figure 5). SOD (r^2^ = 0.963), FLA (r^2^ = 0.729), DPPH (r^2^ = 0.764), CHR (r^2^ = 0.935), a* (r^2^ = 0.912), and b* (r^2^ = 0.949) were positively correlated to PC1; whereas, PPO (r^2^ = −0.878), GR (r^2^ = −0.874), L* (r^2^ = −0.901), and HUE (r^2^ = −0.802) were negatively correlated with the first principal component. CAT (r^2^ = 0.715), APX (r^2^ = 0.892), MDHAR (r^2^ = 0.912), DHAR (r^2^ = 0.777), POL (r^2^ = 0.877), and AA (r^2^ = 0.952) showed a positive correlation to PC2. Ascorbic acid, POL, ANT and FLA contents, APX, antioxidant (DPPH), and CAT activities were positively correlated to F2, whereas MDHAR and DHAR activities were positively correlated to F3. Furthermore, L*, PPO, and GR activities were positively correlated to F4. 

The Pearson matrix underlined the inter-correlations among all analyzed traits (Table S1; Supplementary Materials). CAT showed a positive correlation to APX (r^2^ = 0.877; *p* ≤ 0.01) (Figure 5), because both are involved in the detoxification of H_2_O_2_. APX is positively correlated with MDHAR (r^2^ = 0.738; *p* ≤ 0.01) and AA (r^2^ = 0.961; *p* ≤ 0.01), both being involved in the glutathione-ascorbate cycle (Figure 3). Other positive correlations among MDHAR, DHAR, and GR (r^2^ = 0.926; r^2^ = 0.695; *p* ≤ 0.01) were found, respectively. POL was positively correlated with all tested non-enzymatic antioxidants such as FLA, DPPH, ANT, and AA (r^2^ = 0.859; r^2^ = 0.807; r^2^ = 0.990; r^2^ = 0.863; *p* ≤ 0.01), respectively, due to the massive stress response induced by flower development, and negatively with PPO (r^2^ = −0.693; *p* ≤ 0.01), because polyphenoloxidase plays an important role in the browning reaction determining the oxidation of polyphenol compounds. Several studies demonstrated that multivariate data analysis is a valid tool to underline similarities and differences between all analyzed traits [56,57]. 

## 4. Conclusions

Edible flowers have attracted more attention due to the high content of phytochemicals with health benefits. Feijoa flowers collected at different development stages showed great variations with respect to physicochemical and qualitative features. The non-enzymatic antioxidant defense system, characterized by polyphenols, anthocyanins, and ascorbic acid, showed the highest values in feijoa petals harvested at the F2 stage. Furthermore, we observed several changes in both enzymatic antioxidant defense system and ascorbate-glutathione cycle involved in the regulation of flower development. Based on our results, we defined F2 as the optimal stage to harvest feijoa flowers in order to ensure a better retention of the antioxidant components.

More efforts should focus on the new processes and technologies aimed at preserving feijoa flowers to guarantee longer shelf-life, evaluating also the antioxidant system involved in their senescence. 

## Figures and Tables

**Table 1:** Physicochemical parameters of feijoa petals at different flowering stages (TA: Titratable acidity; RS: Reducing sugars; L*: Lightness; CHR: Chroma; HUE: Hue angle).

Flowering Stages	TA	RS	L*	a*	b*	CHR	HUE
**F1**	0.12 ± 0.02 (e)	3.06 ± 0.06 (a)	28.82 ± 0.27 (a)	46.06 ± 0.41 (e)	20.07 ± 0.15 (d)	50.24 ± 0.41 (e)	23.54 ± 0.17 (b)
**F2**	0.08 ± 0.005 (d)	3.87 ± 0.27 (b)	30.30 ± 0.02 (c)	45.31 ± 0.07 (d)	20.62 ± 0.06 (e)	49.78 ± 0.07 (d)	24.47 ± 0.06 (c)
**F3**	0.07 ± 0.004 (c)	4.10 ± 0.09 (c)	29.93 ± 0.03 (b)	37.64 ± 0.03 (c)	7.14 ± 0.03 (c)	38.31 ± 0.03 (c)	10.74 ± 0.05 (a)
**F4**	0.05 ± 0.003 (b)	4.45 ± 0.12 (d)	31.65 ± 0.05 (e)	34.38 ± 0.06 (b)	−2.93 ± 0.04 (a)	34.50 ± 0.06 (b)	355.13 ± 0.07 (d)
**F5**	0.04 ± 0.005 (a)	4.84 ± 0.03 (e)	31.47 ± 0.04 (d)	24.72 ± 0.07 (a)	−1.47 ± 0.06 (b)	24.77 ± 0.07 (a)	356.59 ± 0.14 (e)

Petals begin to open: Reddish stamens and carpel visible—F1; petals continue to open: Anthers and filaments with dark reddish color—F2; petals fully open: Anthers, filaments, and carpel with dark reddish color—F3; anthers cream-colored and carpel reddish/Carpel taller than anthers—F4; anthers acquire a black color after dehiscence—F5. Means followed by the same letter do not differ significantly at *P* = 0.05 (Duncan Test).

## Supplementary Materials

The following are available online at https://www.mdpi.com/2304-8158/9/1/95/s1, Table S1: Pearson Matrix.
