# Supplementary material for: Evaluation of Antioxidant Systems and Ascorbate-Glutathione Cycle in Feijoa Edible Flowers at Different Flowering Stages
Source: Foods. 2020 Jan 16;9(1):95. doi: 10.3390/foods9010095 (PMC7022405; doi:10.3390/foods9010095)
Supplement: Supplementary file 1 [file foods-09-00095-s001.pdf]

Table 1S Pearson's correlation matrix between all analysed traits (POL: total polyphenol content; AA: ascorbic acid content; FLA: flavonoids; ANT: anthocyanins; DPPH: antioxidant activity SOD: superoxide dismutase, CAT: catalase, PPO: polyphenoloxidase, APX: ascorbate peroxidase, MDHAR: monodehydroascorbate reductase, DHAR: dehydroascorbate reductase and GR: glutathione reductase, L*: lightness, CHR: Chroma and HUE: hue angle).

	**CAT**	**SOD**	**APX**	**PPO**	**MDHAR**	**DHAR**	**GR**	**POL**	**FLA**	**DPPH**	**ANT**	**AA**	**L***	**a***	**b***	**CHR**	**HUE**
**CAT**	1	,449	,877**	-,691**	,436	,135	-,238	,754**	,742**	,946**	,802**	,850**	-,458	,750**	,812**	,771**	-,759**
**SOD**		1	-,013	-,893**	-,405	-,482	-,913**	,330	,748**	,668**	,330	,037	-,897**	,770**	,886**	,807**	-,704**
**APX**			1	-,342	,738**	,472	,224	,725**	,496	,723**	,777**	,961**	-,025	,378	,443	,394	-,457
**PPO**				1	-,043	,063	,653**	-,693**	-,950**	-,884**	-,682**	-,424	,854**	-,720**	-,940**	-,767**	,837**
**MDHAR**					1	,926**	,695**	,660**	,239	,331	,632	,769**	,205	-,187	-,038	-,185	-,208
**DHAR**						1	,757**	,568*	,152	,096	,512	,563*	,309	-,475	-,247	-,462	,009
**GR**							1	-,027	-,492	-,415	-,057	,205	,716**	-,692**	-,719**	-,724**	,435
**POL**								1	,859**	,807**	,990**	,863**	-,339	,303	,605*	,357	-,587*
**FLA**									1	,899**	,858**	,619*	-,664**	,573*	,868**	,637	-,724**
**DPPH**										1	,827**	,743**	-,670**	,783**	,927**	,817**	-,864**
**ANT**											1	,902**	-,293	,339	,627	,396	-,554*
**AA**												1	-,040	,300	,461	,334	-,438
**L***													1	-,783**	-,832**	-,785**	,883**
**a***														1	,899**	,995**	-,829**
**b***															1	,932**	-,863**
**CHR**																1	-,823**
**HUE**																	1
